# On the selection of appropriate distances for gene expression data clustering

**DOI:** 10.1186/1471-2105-15-S2-S2

**Published:** 2014-01-24

**Authors:** Pablo A Jaskowiak, Ricardo JGB Campello, Ivan G Costa

**Affiliations:** 1 Institute of Mathematics and Computer Sciences, University of São Paulo, São Carlos - SP, Brazil; 2 Center of Informatics, Federal University of Pernambuco, Recife - PE, Brazil; 3 IZKF Computational Biology Research Group, Institute for Biomedical Engineering, RWTH Aachen University Medical School, Aachen, Germany

**Keywords:** distance, similarity, dissimilarity, correlation, proximity, clustering, gene expression, microarray, cancer, time-series

## Abstract

**Background:**

Clustering is crucial for gene expression data analysis. As an unsupervised exploratory procedure its results can help researchers to gain insights and formulate new hypothesis about biological data from microarrays. Given different settings of microarray experiments, clustering proves itself as a versatile exploratory tool. It can help to unveil new cancer subtypes or to identify groups of genes that respond similarly to a specific experimental condition. In order to obtain useful clustering results, however, different *parameters *of the clustering procedure must be properly tuned. Besides the selection of the clustering method itself, determining which distance is going to be employed between data objects is probably one of the most difficult decisions.

**Results and conclusions:**

We analyze how different distances and clustering methods interact regarding their ability to cluster gene expression, i.e., microarray data. We study 15 distances along with four common clustering methods from the literature on a total of 52 gene expression microarray datasets. Distances are evaluated on a number of different scenarios including clustering of cancer tissues and genes from short time-series expression data, the two main clustering applications in gene expression. Our results support that the selection of an appropriate distance depends on the scenario in hand. Moreover, in each scenario, given the very same clustering method, significant differences in quality may arise from the selection of distinct distance measures. In fact, the selection of an appropriate distance measure can make the difference between meaningful and poor clustering outcomes, even for a suitable clustering method.

## Background

Microarray development has enabled researchers to gather huge amounts of data from the most diverse biological phenomena. A single microarray is capable of determining expression levels for virtually all the genes of a particular biological sample of interest. Once combined, related microarray experiments give rise to what is usually referred to as gene expression data, a highly dimensional dataset with measurements over thousands of genes and few biological samples (microarrays). Obtaining the data is, however, only the first step towards the laborious path that comprehends its analysis.

To transform gene expression data into knowledge, efficient and effective computational methods are required. Methods from Data Mining, Machine Learning, and Statistics have been applied since the birth of the gene expression data analysis field [1-3]. A frequently used method is clustering, as its unsupervised nature, allows the creation of new hypothesis from gene expression data. In the gene expression data domain clustering has two distinct applications. The first one is obtained when biological samples are clustered together. In this application scenario the main objective is to detect previously unknown clusters of biological samples, which are usually associated with unknown types of cancer [4]. Since the seminal work presented by Golub et al. [5], the clustering of cancer samples has become a routine in high throughput cancer studies, such as [6-9]. Once cancer signatures are identified on a genomic level, specific drugs can be developed, improving treatment efficacy while reducing its side effects.

The second clustering application concerning gene expression data is found when genes that show similar expression patterns are clustered together [2,10-12]. In this particular application scenario, different microarray experiments are usually performed with the same biological sample in different time instants for a given process of interest, e.g., cell cycle. Such experiments have also been employed to study cell response to different types of stress conditions, e.g., starvation, and to drug treatments, e.g., [13,14]. Usually such time series are measured over few time points, have distinct time scales and frequencies. The clustering of gene time-series can help researchers to determine genes that have similar function or are co-regulated, just to mention a few of its applications [10,11,15].

Taking into account the peculiarities of each one of the aforementioned scenarios, several clustering methods have been proposed for the problem of tissue clustering, e.g., [16-19], and short gene time-series data, e.g., [15,20-22]. Moreover, classical methods from the clustering literature have been borrowed and employed with success to analyze gene expression microarray data, including, but not limited to, hierarchical methods [23], k-means [24], and k-medoids [25]. Given the plethora of clustering methods, a user usually faces the question: which clustering method is more suited to my analysis? To answer such a question numerous theoretical and empirical studies have been conducted [4,10,11,26-30].

There is no doubt that a suitable clustering method is needed to achieve good quality clustering results. However, selecting a clustering method is one of several *parameters *that comprise the clustering procedure. Provided that most clustering methods are based on distance calculations, i.e., clusters are determined based on distances between objects, selecting the distance between pairs of objects to be employed by the clustering method is at least as important as selecting the clustering method itself [1,23,31-33]. Yet, the distance *parameter *has often been overlooked in what concerns the analysis of gene expression data, as pointed by [1,31,32,34]. If on one hand diverse studies addressed the issue of clustering method selection, on the other hand just a few tried to provide guidelines regarding the selection of distances for gene expression data. Thus, when the question "which distance measure is more suited to my analysis?" is asked by the user, there is still no precise answer to this date.

In view of gene expression data, objects are deemed similar if they exhibit trend or shape similarity [15]. Although this somehow limits the number of choices from the whole universe of distance measures, there is still a considerable variety of measures capable of identifying trend similarity available in the general clustering literature. Additionally, some distances have been specifically introduced aiming the clustering of gene time-series, e.g., [15,35-37], taking into account its temporal characteristic. Despite the variety of distances available for gene expression data clustering, few previous works have addressed the problem of distance evaluation.

Theoretical reviews highlighting the importance of selecting appropriate distances for the clustering of gene expression data have been conducted by [10] and [38]. Although such studies opened venues for further investigation on the subject of distance measures, they do not provide any guidelines on how to select a particular one. Besides presenting and reviewing several different distance measures these studies do not suggest which distance measures should be preferred, favored, or avoided.

One of the first empirical studies concerned with the comparison of distances for gene expression data was conducted by [39]. The authors focused on the comparison of three different distances for the clustering of short gene time-series. Measures were compared considering three different datasets. In [40] the authors considered five different distance measures during the comparison of clustering methods for gene time-series clustering. Although [39,40] focus specifically on the clustering of gene time-series data, neither consider distance measures that were specifically proposed to this scenario. In fact, most distance measures specifically designed for gene time-series were introduced after such studies.

Considering the clustering of cancer samples, different distances were evaluated by [4], [30], and [41]. In [4] the authors consider the largest collection of datasets so far, 35 datasets from both cDNA and Affymetrix microarrays. In both [4] and [30], however, the authors are primarily interested in the comparison of clustering methods rather than the distances themselves. Furthermore, we note that even in the study performed by [41], in which the authors are mainly concerned with the evaluation of different distance measures, only a small number of different distances is taken into account.

Distance measures are also compared by [42] and [43]. It is worth noticing, however, that in these two studies only a small set of both distances and datasets are considered. Furthermore, the authors take into account, without any distinction, both the clustering of cancer samples and the clustering of gene time-series, which are fairly different problems by nature. In addition, distance measures specifically designed for gene time-series data are not considered in these studies. Given that two quite different application scenarios are combined into a single analysis we believe that conclusions from these two works may be biased and should thus be examined with care.

The first large study analyzing different distances regarding gene expression microarray data was performed by [34]. This was the first comprehensive empirical study that evaluated distance measures for both gene time-series and cancer data independently. Differently from previous studies such scenarios were considered *separately *for analysis, given their different characteristics. The authors also reviewed and evaluated, for the first time, distances that were explicitly introduced for short gene time-series clustering.

This paper is complementary to our previous work [34]. There, we evaluated distances *without *applying a clustering method. This was possible due to the concept of *intrinsic separation ability*, which compares directly a distance measure against a desired ground truth solution, i.e., a reference partition. Therefore, there is no guarantee that the distance measures that provided good performance in [34] are going to behave well when employed in conjunction with a particular clustering method. In this paper we further explore the conjectures raised in [34], filling the gap left by this particular work. Along with [34] our work establishes a solid guidance regarding the selection of distances for gene expression data clustering.

## Results

We take into account 15 different distance measures. From this total, 6 are correlations, namely, Pearson (PE), Goodman-Kruskal (GK), Spearman (SP), Kendall (KE), Weighted Goodman-Kruskal (WGK) and, Rank-Magnitude (RM). We also include in our analysis four "traditional" proximity measures, i.e., Cosine similarity - adapted as distance (COS), Euclidean distance (EUC), Manhattan distance (MAN) and Supreme distance (SUP), the last three being special cases of the Minkowski Distance. Finally, we consider 5 measures that were tailored for clustering short gene time-series, namely, Jackknife (JK), Short Time-Series Dissimilarity (STS), Local Shape-based Similarity (LSS), YS1, and YR1. From now on, we refer to all the aforementioned measures by the term *distances*, since all of them are adapted to distances. For their definitions, please refer to the Methods Section.

We evaluate the aforementioned measures with four different clustering methods commonly employed to the clustering of gene expression data [4,11,30,44,45], i.e., k-medoids (KM) [25] and three hierarchical clustering methods [23]: Complete-Linkage (CL), Average-Linkage (AL) and, Single-Linkage (SL). At this point, it is important to explain our preference for k-medoids over the more popular k-means. Considering k-means and the well-known Euclidean distance, the arithmetical mean of the objects that belong to a cluster defines its centroid. For distance measures other than Squared Euclidean distance, however, the centroid calculation must be redefined to maintain k-means optimization and convergence proprieties [46]. To avoid convergence problems, we use k-medoids, a counterpart of k-means in which the centroid is replaced by the medoid (most representative object in the cluster).

Our analysis is performed on a total of 52 real microarray datasets, comprising both the clustering of gene time-series (17 datasets) and the clustering of cancer samples (35 datasets). Datasets from gene time-series and cancer samples come from two benchmark sets, introduced in [34] and [4], respectively (see the Methods Section for details). Different evaluation settings are considered to provide a broad view of the general performance of the distances under evaluation. Such scenarios are intimately related to the type of data under evaluation, as we discuss in the following.

For the cancer datasets the number of clusters of each dataset in known *a priori*, as well as the cluster memberships for objects in these datasets, i.e., we have a ground truth. In such a case, one can employ measures such as the Adjusted Rand Index (ARI) [23,47]. This index indicates the degree of concordance between a partition obtained with the pair clustering method-distance measure and the reference partition from the dataset in question.

Note, however, that for gene time-series data no class labels are available. That is, we do not know *a priori *cluster memberships for the objects in these datasets. In fact there are a few labeled or synthetic gene expression time-series datasets proposed in the literature. We note, however, that these datasets have a small number of genes and do not represent a real scenario in which one has at least one thousand genes to cluster. In this case, a different evaluation procedure is needed. For instance, one can evaluate results based on their agreement with available biological knowledge, e.g., from the Gene Ontology [48], as we describe during the discussion of the gene time-series clustering results. We summarize in Table 3 which evaluation scenarios are considered for each type of data (# denotes *number*). Details for each evaluation scenario are given along with the discussion of its results.

**Table 1:** Summary of the cancer benchmark data employed in our evaluation.

	Name	*nc*	*no*	*nf*
	*armstrong-v1*	2	72	1081
	*chowdary*	2	104	182
	*golub-v1*	2	72	1877
	*gordon*	2	181	1626
	*laiho*	2	37	2202
Affymetrix	*nutt-v2*	2	28	1070
	*nutt-v3*	2	22	1152
	*pomeroy-v1*	2	34	857
	*shipp*	2	77	798
	*singh*	2	102	339
	*west*	2	49	1198
	*yeoh-v1*	2	248	2526
	*armstrong-v2*	3	72	2194
	*dyrskjot*	3	40	1203
	*golub-v2*	3	72	1877
	*nutt-v1*	4	50	1377
	*bhattacharjee*	5	203	1543
	*pomeroy-v2*	5	42	1379
	*yeoh-v2*	6	248	2526
	*su*	10	174	1571
	*ramaswamy*	14	190	1363

	*alizadeh-v1*	2	42	1095
cDNA	*chen*	2	180	85
	*bittner*	2	38	2201
	*bredel*	3	50	1739
	*lapointe-v1*	3	69	1625
	*liang*	3	37	1411
	*alizadeh-v2*	3	62	2093
	*tomlins-v2*	4	92	1288
	*alizadeh-v3*	4	62	2093
	*garber*	4	66	4553
	*khan*	4	83	1069
	*lapointe-v2*	4	110	2496
	*risinger*	4	42	1771
	*tomlins-v1*	5	104	2315

Columns display name of the data, number of clusters (*nc*), number of objects (*no*) and, number of features (*nf *), respectively.

**Table 2:** Summary of the time-series benchmark data employed in our evaluation.

Name	Source	*noo*	*nfo*	*nf*
*1M sorbitol*		1030	6152	7
*diauxic shift*		1016	6152	7
*complete DTT*		962	6152	7
*heat shock 2*		999	6152	7
*1.5mM diamide*		1038	6152	8
*2.5mM DTT*	Gasch *et al*. (2000)	991	6152	8
*heat shock 1*		988	6152	8
*1mM menadione*		1050	6152	9
*constant 32nM H2O2*		976	6152	10
*nitrogen depletion*		1011	6152	10
*YPD 2*		1022	6152	10
*YPD 1*		1011	6152	12

*elutriation*		935	6178	14
*cdc 28*		1044	6178	17
*alpha factor*	Spellman *et al*. (1998)	1099	6178	18
*cdc 15*		1086	6178	24

*sporulation*	Chu *et al*. (1998)	1171	6118	7

Columns display name of the data, source, number of objects originally in the dataset (*noo*), number of filtered objects (*nfo*) and, number of features (*nf *), respectively.

**Table 3:** Evaluation scenarios applied to each type of data.

	Data Type	
	
Evaluation Scenario	Cancer Sample	Gene Time-Series
Fixed # of Clusters	✓	-
Variable # of Clusters	✓	-
Estimated # of Clusters	✓	✓
Robustness to Noise	✓	-

Finally, our primary interest lies on the comparison of distances rather than on the assessment of clustering methods. Note, however, that distance measures are always employed with a clustering method and not as a single entity. It is clear, thus, that the clustering method introduces a bias that is *combined *with the bias provided by each distance. Therefore, during our evaluation we choose to comparatively evaluate distances solely when considering the very same clustering method, unless clearly stated otherwise. This way, we first set the bias of the clustering method, providing a common ground for which the biases of different distances can be taken into account.

## Cancer sample clustering

In the following we present results for cancer datasets.

### Fixed number of clusters

In the first evaluation scenario, we generate partitions containing the same number of clusters as defined by the reference partition, i.e., the original labeling of each dataset. Resulting partitions are then compared based on their Adjusted Rand Index (ARI) [23,47] values, which evaluate the capability of each distance in recovering partitions in conformity with the structure defined in the ground truth. ARI is defined and described in the Methods Section.

**Figure 1:**
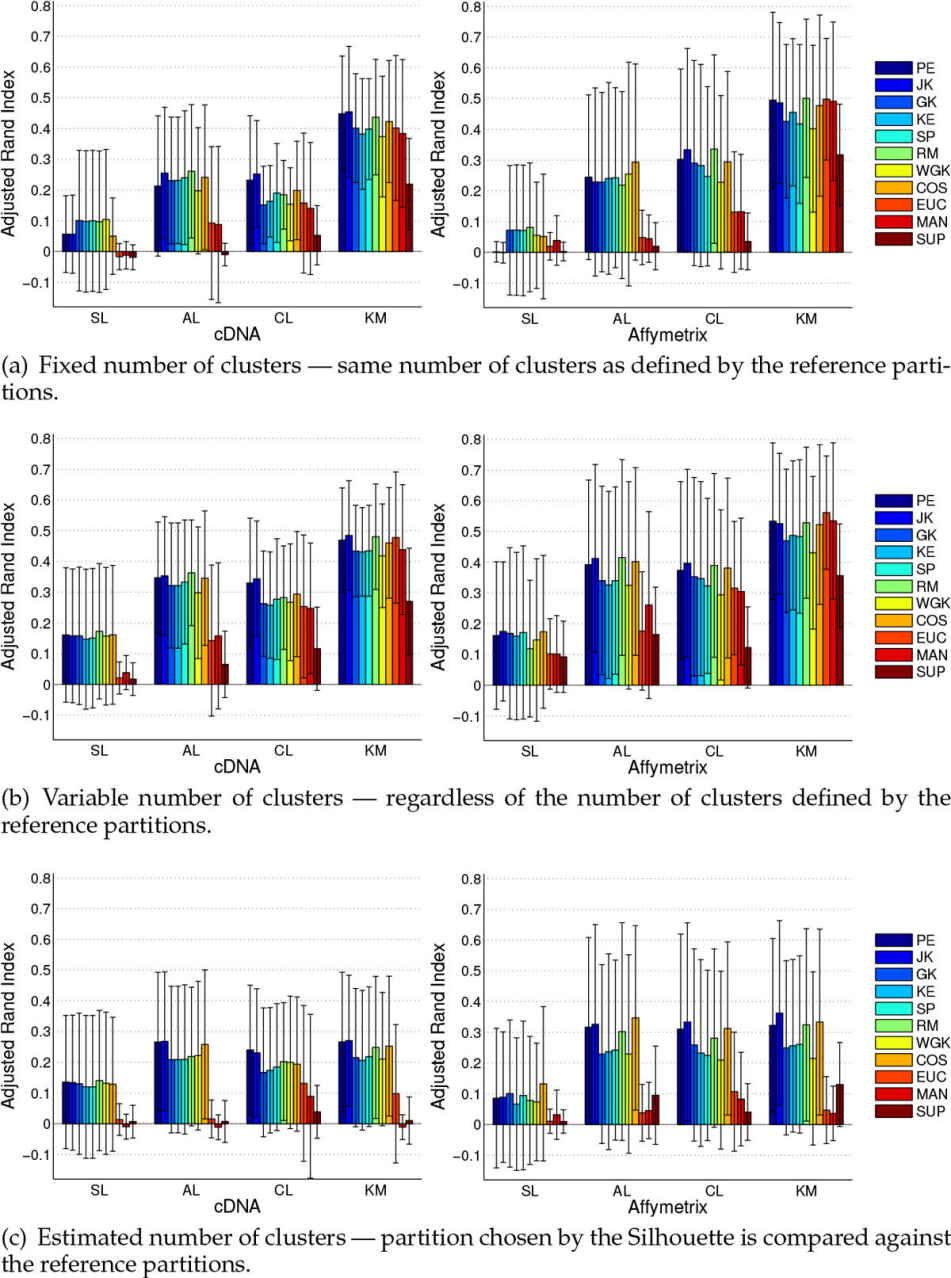
**Cancer Datasets Results: Class recovery obtained for cancer datasets regarding the three evaluation scenarios under consideration, subfigures (a), (b), and (c)**. Bars display mean results for each pair of clustering method and distance function in different types of datasets: cDNA (left) and Affymetrix (right).

Results for this scenario are presented in Figure 1(a). Considering the Average-Linkage clustering method and Affymetrix data, practically all the correlation coefficients employed display similar mean ARI results, whereas the best results are provided by COS. For cDNA datasets, JK and RM present the best mean results, followed by COS. Still regarding this type of data, WGK and PE provide the worst results among the correlation coefficients. For both data types, distances that are based solely on ranks, namely, GK, SP and, KE, present similar behavior among themselves, whereas "traditional" distances provide the worst results.

**Figure 2:**
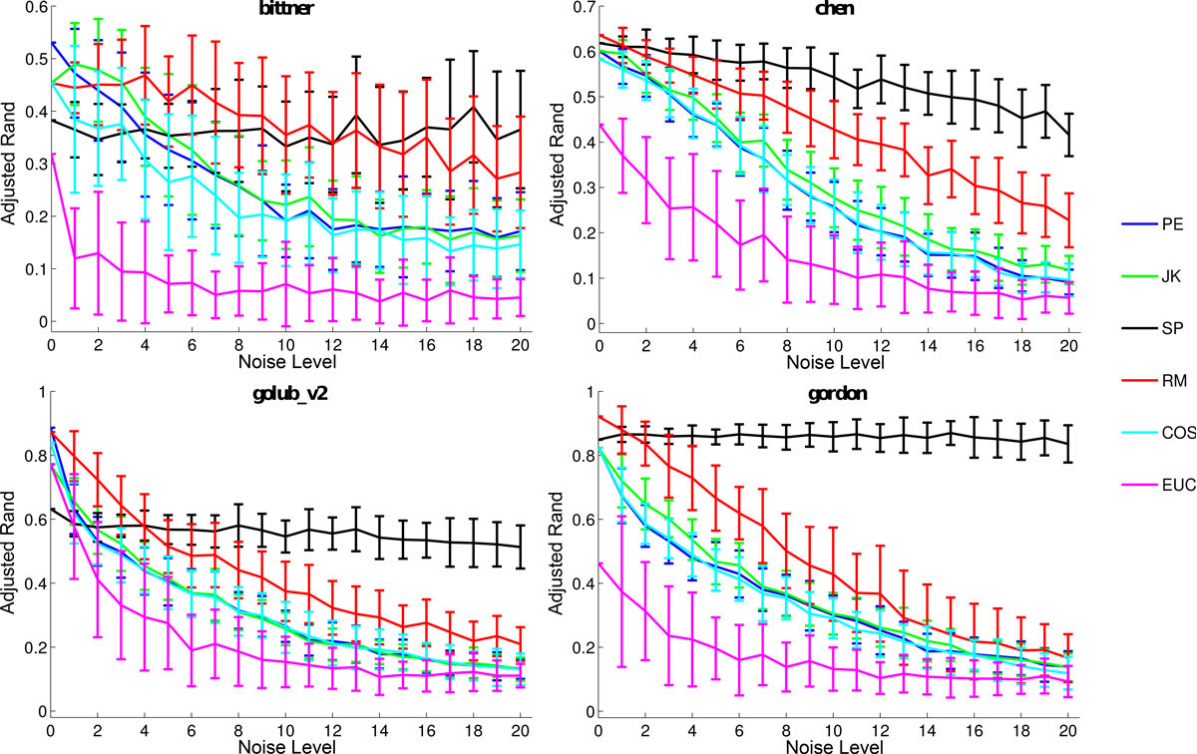
**Robustness to Noise for Cancer Datasets: ARI values for different noise levels (%) regarding PE, JK, SP, RM, COS and EUC**. Plots correspond to the mean ARI values for runs performed in 100 different noisy datasets with the same amount (%) of noise points. Bars account for standard deviations.

For Complete-Linkage and k-medoids clustering methods JK, RM and PE stand out among the other correlation coefficients, except for cDNA datasets with Complete-Linkage, for which RM shows poorer results than JK and PE. Regarding correlation coefficients that take into account only ranks, both KE and GK, which are measures not extensively adopted in gene expression analysis, show in particular cases superior mean results when compared to the also rank-based SP. Among the "traditional" distances, SUP provides the worst results. For the k-medoids method, COS, EUC and MAN provide competitive but slightly worse results than the top distances (RM, JK and PE).

As reported in [4] and [41], the Single-Linkage clustering method leads to the poorest recovery rates among the clustering methods employed. Our results support and reinforce the results presented in [4,41], because even with the use of different distance measures, the Single-Linkage method clearly does not stand as a good choice for the sample clustering scenario.

We applied statistical tests (see Methods Section for description) in separate for each clustering method to detect which distances provided statistically superior results regarding their ARI values. For both cDNA and Affymetrix, considering AL, CL, and KM clustering methods, PE, JK, and RM provide better results than SUP in virtually all cases. For Single-Linkage no statistical differences are suggested.

### Variable number of clusters

In the second evaluation scenario we choose for further comparison partitions that provide the best ARI values, regardless of their number of clusters. For a given dataset we generate partitions within the interval $\left[2,\left\lceil \sqrt{o}\right\rceil \right]$, where *o *stands for the number of objects. Note that partitions with number of clusters different from those found in the reference partition may, in certain cases, contain more natural clusters than those found in a partition with the "right" number of clusters, see, e.g., [49].

We depict in Figure 1(b) results for such evaluation scenario. In comparison to the former scenario, there is an improvement in the results for all the pairs of clustering methods and distances. This behavior is in agreement with the assumption that a partition with the "wrong" number of clusters may be better than one partition with the "right" number of clusters [49]. Based on this fact, we believe that ARI values are more important than the actual number of clusters in the partitions, and choose not to analyze the latter.

For Average-Linkage, RM, COS, PE and, JK provide the best results for both data types. All correlations based on ranks, i.e., KE, SP and GK, provide similar results among themselves. The worst results are displayed by SUP, MAN and EUC. Note that even the correlation that provided the worst mean results (WGK) stands as a better alternative than the three "traditional" distances.

Regarding Complete-Linkage clustering method, for cDNA data JK and PE provide the best mean results. Still for this kind of data, all the other distances provide quite similar mean results, except for SUP, which provides the worst mean results. For Affymetrix, JK, RM and COS stand out as the best distances. Once again, SUP provides the worst mean results.

When considering the k-medoids clustering method, RM, JK, PE, COS and EUC provided similar mean results among themselves. For Affymetrix data, MAN performs close to the aforementioned distances. Correlations based on ranks provide, on average, worse accuracy than previously mentioned distances. Considering only correlation coefficients, WGK provides the worst mean results. Regardless of the kind of data, SUP provides the worst results.

The Single-Linkage clustering method shows the overall worst results, regardless of the distance employed. Indeed, for this particular clustering method, all correlation coefficients display very similar results for cDNA and Affymetrix datasets. In particular, EUC, MAN and SUP provide the worst mean results for the Single-Linkage clustering method.

Statistical evaluation for cDNA and KM suggests difference in favor of RM over WGK. Still regarding cDNA, regardless of the clustering method, all correlations are superior to SUP, whereas for the AL method RM, JK, and PE are superior to MAN and EUC. Regarding Affymetrix the tests suggest that RM, PE (only for KM), and JK (except for KM) are statistically superior to SUP.

### Estimated number of clusters

In this evaluation scenario we simulate a real application in which the user has no knowledge on the number of clusters in the data. For each dataset we generate partitions within the interval $\left[2,\left\lceil \sqrt{o}\right\rceil \right]$, where *o *stands for the number of objects. Differently from the previous scenario, however, the best partition for each pair of cluster method and distance is chosen by the Silhouette criterion [50] -- defined in the Methods Section.

We proceed as follows: (i) the best partition for each pair of clustering method and distance, as chosen by the Silhouette, is selected for comparison; (ii) we compute the Adjusted Rand Index (ARI) for the best partitions, i.e., we compute the ARI for the best partition selected by the Silhouette for each pair of clustering method and distance. In this particular step, we are assessing how good are the partitions selected by the Silhouette in step one, for each pair of clustering method and distance, according to the external criteria; (iii) finally, we compare the ARI values for each of the partitions as computed in step (ii). Note that, differently from the previous two scenarios, class label information is employed *only *to validate the results, i.e., it is *not *employed to select the best partition for each pair clustering method-distance, which is not possible in a real clustering application.

Results are displayed in Figure 1(c). Besides the comparison of the distances themselves, it is quite interesting to observe that k-medoids does not provide, in real applications (as simulated by this scenario), significant differences when compared to hierarchical methods. Note that differences among clustering methods are more evident in the previous evaluation scenarios, regardless of the distance employed. More striking than the previous observation is the fact that, despite the similar behavior shown by clustering methods in this scenario, different distances do provide quite different results (in the remaining of the analysis we do not take into account Single-Linkage, which produced, once again, the worst results, regardless of the distance measure employed).

When considering cDNA datasets, JK and PE show the best overall results, for all the clustering methods. Considering results for Affymetrix datasets, it is reasonable to suggest that four distances provide superior results, namely, JK, RM, COS, and PE. In fact, for Affymetrix data, RM shows very competitive results in comparison to COS, PE and, JK. Correlations based on ranks once again show inferior accuracy with respect to other correlation measures, for both types of data. When compared against other correlations WGK shows, in some cases, smaller differences in accuracy (in the former two scenarios this correlation coefficient produced, in a number of cases, the worst results among all the correlations under evaluation). Finally, SUP, MAN and, EUC appear with the lowest accuracy for all the clustering methods considered.

Statistical evaluation suggests that for AL, regarding cDNA, JK and PE are superior to SUP and MAN, whereas for Affymetrix, SP, JK, COS, and PE are superior to EUC. Considering CL, for both data types JK and PE are superior to SUP. For KM and cDNA data, all correlations and COS provide better results than MAN and SUP, whereas for KM and Affymetrix, RM, JK, and PE provide better results than EUC.

### Robustness to noise

We also perform experiments to evaluate the robustness of distances after noise injection. To perform these experiments we choose four particular datasets, two from cDNA and two from Affymetrix, in which all the distances display the same (or at least close) ARI values regarding the original data, i.e., without any noise addition. In such a manner we believe that an impartial comparison of the distances is possible, given that they behave similarly for the original data, i.e., data with no noise.

We artificially introduce noise in the four selected datasets by: (i) choosing *α*% expression values at random (each point corresponds to the expression level of a pair sample - gene) and; (ii) assigning random values (between the maximum and minimum values from the original data) to such points. We examine *α *values between 1% and 20% with 100 noisy datasets for each *α *value.

Results of such evaluation are shown in Figure 2 (cDNA top and Affymetrix bottom). We analyze results for the distances that displayed a good accuracy (in terms of ARI) in the preceding evaluation scenarios, namely, RM, JK, COS, and PE. Given their popularity, we also show results for SP and EUC.

Regardless of how much noise is introduced in the datasets SP shows the best overall robustness. Given that SP considers solely ranks in its formulation, larger perturbations in the data are needed to cause a decrease in its final accuracy. Although SP is more robust than RM regarding noise, RM shows better overall results when compared against the remaining distances. COS, JK and PE show only small differences from each other. EUC, in such experiments, appears with the worst robustness to noise.

Even though it shows advantages over other measures regarding robustness to noise, SP provides in the previous three evaluation scenarios, worse accuracy (in terms of ARI) than COS, RM, JK, and PE. With this in mind, we believe that RM should be the first choice for cancer data, given that: (i) it is within the best distances in the past evaluations and, (ii) although it is more sensitive than SP in the presence of noise it shows increased robustness when compared to COS, JK and, PE. Overall, RM shows a reasonable balance between robustness in the presence of different levels of noise and accuracy, with respect to ARI.

### Gene time-series clustering

For time series data, we consider only the third evaluation scenario (estimated number of clusters) given that class labels are not available. Performing noise experiments in such datasets is also impractical, due to: (i) lack of class labels, (ii) the type of evaluation employed (pairwise), which makes comparison among measures for different noise levels not straightforward, and (iii) the amount of time required to biologically evaluate all partitions. More exactly, for each dataset we generate partitions within the interval $\left[2,\left\lceil \sqrt{o}\right\rceil \right]$, where *o *stands for the number of objects. The best partition for each pair of cluster method and distance is chosen by the Silhouette criterion [50] -- defined in the Methods Section.

Given that we do not have a reference partition for time-series datasets we cannot employ an external criterion to evaluate the quality of clustering results, i.e., in this case we cannot employ ARI to validate the results. To compare the results obtained with the different pairs of clustering methods and distances, we adopt a heuristic similar to the one used by [21] and [51]. In brief, the evaluation methodology employs information available from the Gene Ontology (GO) [48] to validate clustering results. The validation is performed from a biological point of view, with the best structured knowledge about genes and their relationships available so far (as represented in the GO).

The validation procedure is as follows. For each clustering result we perform a gene enrichment analysis [52] and obtain the respective list of enriched terms that have a *p*-*value ≤ *0.05 within each cluster. The enrichment test is based on the Fisher Exact Test, which indicates if the overlap between genes in a cluster and in a GO term is higher than expected by change [52]. To perform the gene enrichment analysis we use the well-known *GOstat *tool from [52]. For two result lists *r*_1 _and *r*_2_, we count the number of times that *r*_1 _provided enrichments with smaller *p*-*values *than *r*_2 _and the number of times that *r*_2 _provided enrichments with smaller *p*-*value *than *r*_1_, these are then combined as given by Equation (1).

(1) $$\text{Comparison}\;\text{(}r_{1}\text{, }r_{2}\text{)}=\text{log}\left(\frac{\neq \left(r_{1}<r_{2}\right)}{\neq \left(r_{2}<r_{1}\right)}\right)$$

Note that changing the order of the results under comparison (*r*_1_,*r*_2_) or (*r*_2_,*r*_1_) changes only the sign of the result, not its absolute value. For this comparison procedure, positive values mean that *r*_1 _is better than *r*_2_, whereas negative values means the opposite.

In brief, the evaluation procedure for gene time-series data is as follows: (i) the best partition for each pair of clustering method and distance (as chosen by the Silhouette) is selected for further comparison; (ii) we evaluate all pairs of results obtained based on the previous heuristic. Such an evaluation is made on the basis of Equation (1); (iii) finally, we compare the values obtained for all pairs of results from step (ii).

Before comparing the distance measures themselves, we assess the results of clustering methods, regardless of the distance measure adopted. These results are shown in Table 4, which summarizes results for SL, AL, CL and KM regardless of the distance adopted for the 17 gene time-series datasets. In each table cell we show the number of Wins/Ties/Losses for the row method with respect to the column one. Each table cell comprises 3825 pairwise comparisons. For each cell we have two clustering methods, each of which is evaluated with 15 distance measures in 17 datasets, i.e., 15*15*17 = 3825 pairwise comparisons between any two methods. In this scenario the best results are displayed by KM, which is closely followed by AL and CL. These three methods provide quite competitive results among each other, whereas the worst overall results are provided by SL. Based on the poor results displayed by SL, we choose not to further evaluate distances regarding it.

**Table 4:** Wins/Ties/Losses for 15 distances and 17 datasets.

	SL	AL	CL	KM
SL	--	531/370/2924	378/384/3063	385/323/3117
AL	2912/406/507	--	1903/93/1829	1710/80/2035
CL	3063/386/376	1821/106/1898	--	1803/17/2005
KM	3117/323/385	2032/80/1713	2001/18/1806	--

**Figure 3:**
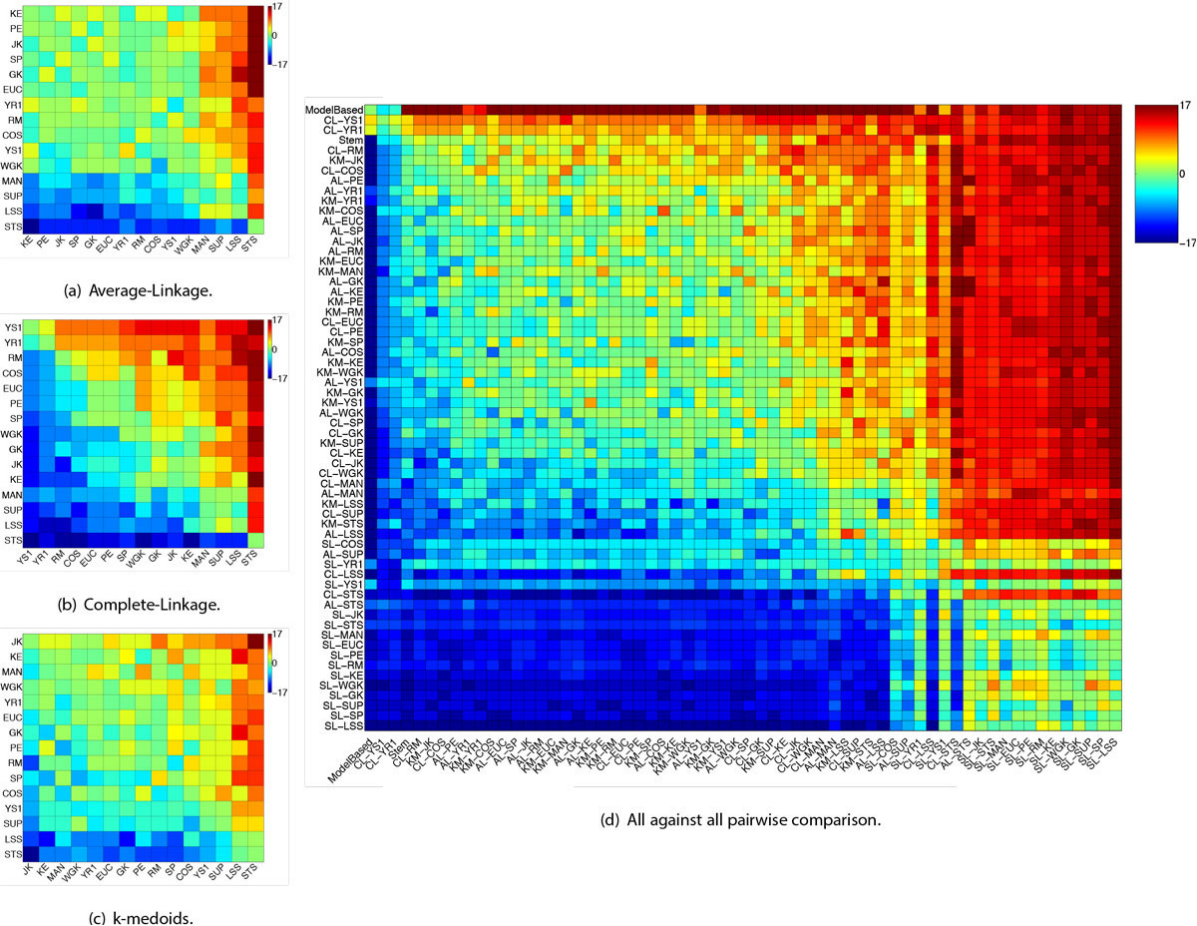
**Gene Time-Series Results: Results for gene time-series data**. Figures (a), (b) and (c) depict pairwise comparison of distances for each clustering method. Figure (d) depicts an all against all pairwise comparison. Each cell account for the number of datasets in which the method from the row obtained a better enrichment than the method from the column. The "hotter"/"colder" the cell the better/worst is the row method in comparison to the column one.

Figure 3(a) depicts results for AL. For this method JK, PE and KE displayed similar results, providing better enrichments than the remaining measures in 71% of the cases under comparison. For AL, none of the measures is consistently better than the others, with different measures appearing as the top ones, depending on the dataset. It is interesting to note that LSS and STS, two measures specifically proposed for the gene clustering scenario, figured as the worst choices (alongside SUP and MAN).

Results for CL are shown in Figure 3(b). For CL, differences among distance measures become more evident. YR1 and YS1, which are tailored for short gene time-series have the best enrichments in 87% and 94% of the evaluated cases. Another distance that showed good results for CL was RM, which provided better enrichments than the other measures in 80% of the cases. These results are better than the ones produced by distances commonly employed for gene clustering, such as PE, EUC, and SP, which provided better results than other distances in 72%, 70% and 65% of the cases, respectively.

We show in Figure 3(c) evaluation results regarding KM. For this clustering method JK provided the best results, showing better enrichments than other distance measures in 77% of the cases under comparison, which is 12% above those found with the second ranked measure (KE). Good results were also shown by MAN, which performed better than other distances in 60% of the cases under comparison. It is worth noting that popular distances in the gene expression clustering literature, namely, SP, EUC and, PE displayed inferior results to at least five other distances under evaluation.

Statistical evaluation was conducted independently for each clustering method. For AL and CL, all measures (except for MAN and SUP) provided better results than LSS and STS. Considering CL alone, YR1, YS1, and RM also displayed better results than MAN and SUP. For KM, all measures (except SUP) provided better results than LSS whereas JK showed better results than STS.

To present an overview of clustering methods and distance measure pairs we conducted an all against all pairwise comparison shown in Figure 3(d). There, we take into account the pair clustering method-distance measure to include both biases in the comparison. To give an idea about the general quality of the results found we also include two clustering methods proposed for clustering of gene time-series, i.e., Stem [53] and Model Based clustering [20]. Regarding Stem, the number of clusters is automatically determined, so we select for comparison the significant clusters it finds. Considering Model Based clustering, the Bayesian Information Criteria (BIC) [54] statistics indicates the number of clusters.

As one might expect, Stem and Model based figured among the top results for all 17 datasets. It is worth noticing that CL, when employed with YS1 and YR1 distance measures produced, in general, better enrichments than Stem and in some cases Model Based. From this comparison it is possible to note that for a particular clustering method, the choice of an appropriate distance measure may provide the difference between an average result and a result close (or better) than those produced by state of the art clustering methods, such as Stem and Model Based.

Note that although the clustering method plays an important role to the clustering outcome, selecting an appropriate distance can significantly enhance its final performance (in terms of clustering quality). To make this clearer, let us take a careful look at Figure 3(d), more specifically at the results produced by CL. For this clustering method, the worst results are obtained in conjunction with STS distance. In fact, results for CL employing STS are as bad as results provided by the SL method, the worst overall clustering method. However, when CL is employed with YR1 or YS1 one can get results as good as (or better) than those obtained with Stem and Model Based clustering. Note that although we are taking CL as an illustrative example, this observation also holds for other reasonable clustering methods, i.e., KM and AL (SL is an exception given the poor quality of its results no matter the distance used).

## Discussion

One of the first observations that should be made is that the choice of distances is application dependent. Although the problem of clustering gene expression data is sometimes considered to be a unique application scenario, this is clearly not the case. As a matter of fact, distinct distance measures stood out for the two different applications under evaluation, i.e., the clustering of cancer samples and the clustering of gene time-series data. Considering our results, it is fair to say that some general trends were observed. We discuss such trends in the sequel.

### Cancer sample clustering

For this type of data Jackknife and Pearson displayed, in most of the cases, the best accuracy in terms of ARI. Cosine also figured amongst the best measures. It is important to note here that Jackknife has quadratic computational complexity, in contrast to linear time complexity of Cosine and Pearson. The minor improvements obtained with Jackknife over Cosine and Pearson do not seem to compensate for its computational cost.

Another interesting alternative in this particular scenario is Rank-Magnitude. In addition to the good results provided for cancer datasets, Rank-Magnitude also showed increased robustness to the presence of noise if compared to Jackknife, Cosine, Pearson and Euclidean distance, though it is more sensitive to noise than Spearman. Given that Rank-Magnitude displayed, in general, a better accuracy than Spearman, we believe it is one of the best alternatives for cancer datasets, with a balance between robustness to noise and accuracy, with a reasonably low running time. It is worth noticing that we have detected little influence on the combination of the clustering methods and distance measures in the results. Overall, they are in agreement with the ones presented in [34].

### Gene time-series clustering

YS1 obtained along with Complete-Linkage the best enrichments on gene expression time-series. These results may be due to the fact that both YS1 (along with YR1) combine a correlation coefficient with other information extracted from the series under evaluation, thus providing a comparison based on more information than the ones performed by any of the other measures considered. By internally employing Spearman, YS1 stands out as a better and more robust option than YR1, which is based on Pearson. In this particular scenario, given the small number of features, Jackknife should be be preferred to both Cosine and Pearson, as it provided better enrichments than both in most cases.

It is interesting to note that Local Shape-based Similarity (LSS) and Short Time-Series dissimilarity (STS) provided poor results for all methods, even though they are tailored for the clustering of short gene expression time-series. Regarding LSS, we believe that the short size of the series under evaluation may prevent the measure to find significant time-shifts. In what concerns the poor results displayed by STS, we believe that the measure is hampered by its over-simplistic formulation. We do not recommend the use of Local Shape-based Similarity, Short Time-Series dissimilarity, and "traditional" distances (except for Cosine), given that better distances are available as alternatives to them, as we discussed.

Despite the fact that the overall trends are in accordance with [34], we observe that the combination of clustering methods and distances are important in the time-series scenario. We speculate that the small dimensionally of the time-series problem imposes the need of a better coupling between the biases of the distance measure and the clustering method.

### Remarks on both clustering Scenarios

Given that a reasonable clustering method is selected, one may note that the choice of an appropriate distance measure has major impact in clustering results. By employing different clustering methods, we do not have exactly the same distance measures standing as best choice. This is expected, since each clustering method imposes a different bias (along with the bias of the distance). Therefore, for a particular clustering method a specific set of distances may be more interesting than another. For both the cancer and gene time-series scenarios results are in conformity with the ones presented in [34]. Our study complements, therefore, our previous work by showing that at least for the clustering methods considered here consistent results are observed.

### Remarks on clustering methods

Although our main focus is the performance of different distances it is possible to observe some trends on the behavior of the four particular clustering methods we considered during our analysis. Some trends may also be identified considering the biases of both clustering methods and distance measures together. Regarding cancer datasets, as a first choice, we recommend the use of k-medoids. If the user would like to employ a hierarchical method, Average-Linkage should be preferred over Complete-Linkage. Considering these particular three clustering methods and cancer data, results suggest that Rank-Magnitude, Jackknife (with a higher computational cost), Pearson, and Cosine are the best alternatives, in this order. When considering time-series datasets the scenario is more intricate. While there is no clear indication of the best method, we have empirical evidence suggesting the application of Complete-Linkage with YS1 and YR1. Regarding the use of k-medoids and Average-Linkage, Jackknife provides good results with both clustering methods. Finally, we do not recommend the use of the Single-Linkage clustering method in *any *scenario whatsoever, regardless of the distance employed.

## Conclusions

We conducted a large scale analysis considering distance measures from different classes and their suitability for clustering gene expression microarray data. In total 15 different distances, 4 clustering methods, 4 evaluation scenarios, and a total of 52 datasets were employed. According to our results the scenario under evaluation should be always considered during the selection of the "right" distance. Finally, although results are dependent of the clustering method employed, it is clear that once a reasonable clustering method is selected large differences in quality can arise from the selection of different distances. We believe that our work provides a compendium of distance measures alternatives to field practitioners as well as valuable guidelines regarding their selection.

## Methods

### Distance measures

After selecting a clustering method one usually has to determine which distance will be employed between objects, given that most clustering methods are based on distance calculations [55,56]. In gene expression one usually seeks for similarity in shape or trend between objects [15]. For such a reason, correlation coefficients have been popular choices [3,10]. As a matter of fact, the well-known Spearman and Pearson correlation coefficients, alongside the traditional Euclidean distance, have found great applicability in gene expression, as highlighted by several authors, e.g., [1-3,10,32,34,57]. There is, however, a number of less-known distance measures that remain practically unexplored to this date. Bearing this in mind we describe the 15 distances that we consider for evaluation in this study. We begin by describing 6 correlation coefficients. Afterwards, we review 4 measures which we refer to as *traditional measures*. Finally we review 5 distance that were tailored for clustering short gene time-series.

#### Correlation coefficients

Correlation coefficients are popular choices for clustering microarray data, with values in the [*−*1, 1] interval. Since the sign of the correlation is important for gene expression data, one minus the value of the correlation provides the distance we use for clustering in our experiments (as is usual in the gene expression literature). In the following, both **x **and **y **are sequences of real numbers in the form **x **= (*x*_1_, . . . , *x_n_*) and **y **= (*y*_1_, . . . , *y_n_*).

*Pearson: *Pearson [58], which is given by Equation (2), is probably one of the most popular correlation coefficients in the literature, allowing one to identify linear relationships of variables. Previous studies have reported that Pearson can display sensitivity when the variables have outliers [3,15]. In such cases variables that are not truly similar (i.e., variables that are similar just because they contain outliers) can end up as false positives, i.e., with a large correlation. Its computation is straightforward, with linear running time.

(2) $$PE(x,y)=\frac{\sum_{i=1}^{n}\left(x_{i}-\bar{x})(y_{i}-\bar{y}\right)}{\sqrt{\sum_{i=1}^{n}{\left(x_{i}-\bar{x}\right)}^{2}}\sqrt{\sum_{i=1}^{n}{\left(y_{i}-\bar{y}\right)}^{2}}}$$

*Goodman-Kruskal: *The Goodman-Kruskal [59] correlation coefficient is a rank-based correlation coefficient. In order to introduce such correlation, let us define first three different types of pairs of values with respect to sequences **x **and **y**, namely: concordant, discordant and, neutral pairs. We define as concordant, those pairs of values that obey a same order, i.e., *x_i _< x_j _*and *y_i _< y_j _*or *x_i _> x_j _*and *y_i _> y_j _*. We call discordant all the pairs for which *x_i _< x_j _*and *yi > yj *or *xi > xj *and *yi < yj *. Pairs that are neither concordant nor discordant are defined as neutrals. Based on these three definitions, the Goodman-Kruskal correlation coefficient is provided by Equation (3), for which *P*_+ _and *P_− _*correspond to the total number of concordant and discordant pairs in sequences **x **and **y**. The Goodman-Kruskal correlation has *O*(*n log n*) running time [60].

(3) $$GK\left(x,y\right)=\frac{P_{+}-P_{-}}{P_{+}+P_{-}}$$

*Kendall: *Kendall [61], which is given by Equation (4), is also a rank-based correlation coefficient. It follows the same definitions previously introduced for Goodman-Kruskal. In Equation (4), the denominator accounts for the number of pairs of values in **x **and **y**. From this different normalization Kendall can achieve its maximum values only when the sequences under evaluation have no neutral pairs. It is easy to observe that Kendall has the same time-complexity as Goodman-Kruskal, that is, *O*(*n log n*).

(4) $$KE\left(x,y\right)=\frac{P_{+}-P_{-}}{n\left(n-1\right)/2}$$

*Spearman: *If the values of each sequence are replaced by their respective ranks, the Spearman correlation coefficient is also given by Equation (2). Given that the actual values of the sequences are replaced by their ranks, Spearman tends to be less sensitive to outliers than its counterpart, Pearson [3]. Due to the need of obtaining ranks for the values in each sequence (the sequences need to be sorted) Spearman has a *O*(*n log n*) running time.

*Rank-Magnitude: *In order to correlate sequences with ranks and real values, [60] introduced the measure called Rank-Magnitude, which in its original version is an asymmetric correlation coefficient. Its asymmetric definition is given by Equation (5), for which $min^{rank}=\sum_{i=1}^{n}y_{i}\left(n-i+1\right)$ and $max^{rank}=\sum_{i=1}^{n}iy_{i}$, given that **y **is sorted in increasing order of values.

(5) $$\hat{r}\left(x,y\right)=\frac{2\sum_{i=1}^{n}Rank\left(x_{i}\right)y_{i}-max^{rank}-min^{rank}}{max^{rank}-min^{rank}}$$

Given that gene expression data is symmetric, i.e., we deal only with real values, we use here a symmetric adaption of Rank-Magnitude [41,62], which we call RM for short. Such symmetric version is easily obtained with $RM\left(x,y\right)=\left(\hat{r}\left(x,y\right)+\hat{r}\left(y,x\right)\right)/2$. Note that although such measure is symmetric, it captures both the behavior of ranks and magnitudes of sequences. Both versions of Rank-Magnitude have an *O*(*n log n*) running time.

*Weighted Goodman-Kruskal: *The measure referred to as Weighted Goodman-Kruskal, introduced by [60], also considers in its formulation both magnitudes and ranks of the sequences under evaluation. It is defined by Equation (6), for which ${\hat{\omega }}_{ij}$ is given in Equation (7). From the latter Equation, ${\hat{\omega }}_{ij}^{x}$ and ${\hat{\omega }}_{ij}^{y}$ account for the percentual (signed) difference from the *i*th and *j*th elements in their sequences and are given by Equation (8). Finally, *ω_ij _*is given by Equation (9), where $\omega_{ij}^{x}=sign\left(x_{i}-x_{j}\right)$ and $\omega_{ij}^{y}=sign\left(y_{i}-y_{j}\right)$. Weighted Goodman-Kruskal running time is *O*(*n*^2^).

(6) $$WGK\left(x,y\right)=\frac{\sum_{i=1}^{n-1}\sum_{j=i+1}^{n}{\hat{\omega }}_{ij}}{\sum_{i=1}^{n-1}\sum_{j=i+1}^{n}|\omega_{ij}|}$$

(7) $${\hat{\omega }}_{ij}\left\{\begin{array}{cc} 1 & \text{if}\;{\hat{\omega }}_{ij}^{x}\text{ and }{\hat{\omega }}_{ij}^{y}=0\\ max\left\{\frac{{\hat{\omega }}_{ij}^{\text{x}}}{{\hat{\omega }}_{ij}^{\text{y}}},\frac{{\hat{\omega }}_{ij}^{\text{y}}}{{\hat{\omega }}_{ij}^{\text{x}}}\right\} & \text{if}\;{\hat{\omega }}_{ij}^{x}\;{\hat{\omega }}_{ij}^{y}<0\\ min\left\{\frac{{\hat{\omega }}_{ij}^{\text{x}}}{{\hat{\omega }}_{ij}^{\text{y}}},\frac{{\hat{\omega }}_{ij}^{\text{y}}}{{\hat{\omega }}_{ij}^{\text{x}}}\right\} & \text{if}\;{\hat{\omega }}_{ij}^{x}\;{\hat{\omega }}_{ij}^{y}>0\\ 0 & \text{otherwise} \end{array}\right)$$

(8) $${\hat{\omega }}_{ij}^{x}=\left\{\begin{array}{cc} \frac{x_{i}-x_{j}}{max_{x}-min_{x}} & \text{if}\;max_{x}\neq min_{x}\\ 0 & \text{otherwise} \end{array}\right)$$

(9) $$\omega_{ij}=\left\{\begin{array}{cc} 1 & \text{if }\omega_{ij}^{x}=0\;\text{and}\;\omega_{ij}^{y}=0\\ \omega_{ij}^{x}/\omega_{ij}^{y} & \text{if }\omega_{ij}^{x}\neq 0\\ 0 & \text{otherwise} \end{array}\right)$$

#### Traditional distance measures

In order to provide a broad view regarding distance measures we also review and evaluate "traditional" distances from the clustering literature. We consider four different distance measures, all of which have linear running time, i.e., *O*(*n*).

*Minkowski: *Distances measures known as Manhattan (MAN), Supreme (SUP) and, Euclidean (EUC) are particular cases of the more general Minkowski family of metric distances [23], defined in Equation (10). Such distances are obtained with different configurations of *λ*, in Equation (10). For the three particular cases of the Minkowski distance we consider in this work, i.e., MAN, SUP and, EUC, we have *λ *= 1, *λ *= *∞ *and, *λ *= 2, respectively.

(10) $$Minkowshi\left(x,y\right)={\left(\overset{n}{\underset{i=1}{\sum }}|x_{i}-y_{i}{|}^{\lambda }\right)}^{1/\lambda }$$

*Cosine: *Cosine is a measure similar to the Pearson correlation coefficient [10]. The only difference between these two measures is due to the fact that Pearson considers the mean of each variable, measuring the difference between their angles considering the origin, whereas Cosine does not, measuring thus their difference based on the mean of the variables under comparison. Made such considerations, Cosine is given by Equation (11).

(11) $$cos_{sim}\left(x,y\right)=\;\frac{\sum_{i=1}^{n}x_{i}y_{i}}{\sqrt{\sum_{i=1}^{n}{\left(x_{i}\right)}^{2}}\sqrt{\sum_{i=1}^{n}{\left(y_{i}\right)}^{2}}}$$

Note that Equation (11) defines a similarity. Cosine dissimilarity, or simply *COS*, can be obtained by 1 minus the value produced by Equation (11).

#### Time-series specific measures

In the following distances tailored for short gene time-series are reviewed. Before reviewing such measures let us define the timestamps in which the values of the features for each gene are measured as **t **= (*t*_1_, . . . , *t_n_*).

*Son and Baek dissimilarities: *Although correlation coefficients can identify sequences with the same trend, they are invariant to swaps in values of both sequences, i.e., changing the ordering of features for both sequences does not alter the final correlation value. Considering such a fact [37] propose the use of two measures, called YS1 and YR1, that consider correlation between sequences but also take into account other relevant information from the time-series under comparison (like the position of their maximum and minimum or the agreement among their slopes).

Given that a time-series with *n *features has *n − *1 slopes, the slopes of two time-series can be compared with the use of Equation (12), with Equation (13) providing the definition of *Incl *and  I, in Equation (12), providing 1 for agreement and 0 in the remaining cases. The slope of a given a time-series **x **and a feature number (timestamp) can be readily obtained with Equation (14).

(12) $$A\left(x,y\right)=\overset{n-1}{\underset{i=1}{\sum }}\frac{I\left(Incl\left(x,i\right)=Incl\left(y,i\right)\right)}{n-1}$$

(13) $$Incl\left(a,i\right)=\left\{\begin{array}{cc} 0 & \text{if}\;slope\left(a,i\right)=0\\ -1 & \text{if}\;slope\left(a,i\right)<0\\ 1 & \text{if}\;slope\left(a,i\right)>0 \end{array}\right)$$

(14) $$slope\left(a,i\right)=\frac{a_{i+1}-a_{i}}{t_{i+1}-t_{i}}$$

Along with the slope information previously defined, the authors consider whether the minimum and maximum values of the time-series under comparison happen in the same feature (timestamp). Such concept is defined in Equation (15).

(15) $$M\left(x,y\right)\left\{\begin{array}{cc} 0 & \text{if }max^{t_{\text{x}}}\neq max^{t_{\text{y}}}\text{and }min^{t_{\text{x}}}\neq min^{t_{\text{y}}}\\ 0.5 & \text{if }max^{t_{\text{x}}}=max^{t_{\text{y}}}\text{or }min^{t_{\text{x}}}=min^{t_{\text{y}}}\\ 1 & \text{if }max^{t_{\text{x}}}=max^{t_{\text{y}}}\text{and }min^{t_{\text{x}}}=min^{t_{\text{y}}} \end{array}\right)$$

YS1 and YR1 take into account Equations (12) and (15) alongside information provided from two correlation measures. YS1, which is given by Equation (16), combines previously introduced information with Spearman correlation coefficient, whereas YR1, Equation (17), takes into account the Pearson correlation coefficient. In such Equations Spearman and Pearson are adapted, respectively, in the following forms: *S*(**x**, **y**) = (1 + *SP *(**x**, **y**))*/*2 and *R*(**x**, **y**) = (1 + *P E*(**x**, **y**))*/*2.

(16) $$Y\;S1\left(x,y\right)=\theta_{1}A\left(x,y\right)+\theta_{2}M\left(x,y\right)+\theta_{3}S\left(x,y\right)$$

(17) $$Y\;R1\left(x,y\right)=\theta_{1}A\left(x,y\right)+\theta_{2}M\left(x,y\right)+\theta_{3}R\left(x,y\right)$$

Note that Equations (16) and (17) are weighted summations, for which one should have *θ*_1 _+ *θ*_2 _+ *θ*_3 _= 1. Given the high cost associated with the estimation of such weights [37] we employed fixed values in order to compare such measures. In all our experiments we employed *θ*_1 _= 1*/*4, *θ*_2 _= 1*/*4, and *θ*_3 _= 1*/*2, as in [37]. The running time for the measures is the same as the correlation coefficient that they employ, i.e., it is *O*(*n log n*) for *YS*1 and *O*(*n*) for *YR*1.

*Short Time-Series dissimilarity: *Taking into account the fact that a time-series is composed by *n − *1 slopes (where *n *is the number of feature in the time-series) [36] introduced a measure called Short Time-Series dissimilarity (STS), which is defined in Equation (18). The measure takes into account the time difference between the biological collection os samples (timestamps). In this sense, shorter intervals have greater impact in the final value of the measure. STS has *O*(*n*) running time.

(18) $$STS\left(x,y\right)=\sqrt{\overset{n-1}{\underset{i=1}{\sum }}\left(\frac{y_{i+1}-y_{i}}{t_{i+1}-t_{i}}\right)-{\left(\frac{x_{i+1}-x_{i}}{t_{i+1}-t_{i}}\right)}^{2}}$$

*Jackknife: *The so-called Jackknife correlation coefficient [15] was introduced aiming to reduce the number of false positives caused by Pearson. Such reduction is sought by removing values from both sequences during the computation of the Pearson correlation coefficient. False positive sequences tend to have a high correlation that will vanish when outlier values are removed. Therefore, Jackknife takes as its final correlation value the smaller Pearson correlation value over the sequences considering the removal of all their features, one at each step. The Jackknife correlation coefficient is formally defined in Equation (19). In such Equation, *PE^i^*(**x**, **y**) stands for PE without considering the *i^th ^*feature of both **x **and **y **(*PE*^0 ^(**x**, **y**) accounts for no feature removal).

(19) $$JK\left(x,y\right)=\underset{0\leq i\leq n}{\text{min}}PE^{i}\left(x,y\right)$$

Although it was proposed for short gene time-series clustering, Jackknife can also be employed in other scenarios (note that it only considers feature removal). Due to such a fact we employed it in all our experiments in this paper. It is easy to verify that Jackknife correlation coefficient has *O*(*n*^2^) running time, which can become prohibitive for data with a large number of features (which is the case for cancer data).

*Local Shape-based Similarity: *The measure called Local Shape-based Similarity, introduced by [35] considers the fact that similarities between genes can occur locally, in a subspace of the features from the time-series. The authors also consider the possibility that such local similarities may be transposed in one of the genes. Therefore, the Local Shape-based Similarity seeks for local and transposed alignments in sequences that have a high score. The alignment with highest score is defined as final value of similarity, given that it represents the best local (possibly transposed) similarity between the two time-series. The measure is given by Equations (20) and (21), for which *S*, accounts for the similarity considered between any two size *k *subsequences of **x **and **y**. The authors suggest a *min_k _*of *n − *2 (*n *is the number of features in the original series) [35].

(20) $$LSS\left(x,y\right)=\underset{min_{k}\leq k\leq n}{\text{max}}Similarity_{k}\left(x,y\right)$$

(21) $$Similarity_{k}\left(x,y\right)=\underset{1\leq i,j\leq n+1-k}{\text{max}}S\left(x\text{[}i,i-1+k\right],y\left[j,j-1+k\right])$$

It is important to note that in order to obtain the final value of the Local Shape-based Similarity one has to compute similarities among different sized sequences (for any two sequences of *same *length LSS uses Spearman correlation). Given that the probability of obtaining high similarity values is greater for sequences with smaller sizes, LSS employs such probability rationale in order to obtain its final similarity value. Made such considerations, *S *is defined as the probability associated with the correlation value for the subsequences being compared (which relates to their sizes). Details on such calculations can be obtained in [35]. Local Shape-based Similarity has *O*(*n*^3^) running time, which according to its authors can be decreased if one employs an approximated version [35].

### Datasets

We consider a total of 52 gene expression datasets in our study. These datasets are both from cancer and gene time-series experiments, as we detail in the following.

*Datasets from cancer studies: *We adopt the benchmark set of 35 datasets compiled by [4] in order to evaluate distance measures for the clustering of cancer data. From these datasets, 14 were obtained with cDNA microarrays, whereas 21 were produced with Affymetrix microarrays. Cancer benchmark data is summarized by Table 1. Please, consult [4] for full details regarding this benchmark set.

*Datasets from short gene time-series studies: *For this type of data we adopt the benchmark set of 17 datasets compiled by [34]. All the datasets from this benchmark set, which come from three independent studies involving yeast, i.e., *Saccharomyces cerevisiae*, were produced employing cDNA microarrays. These datasets are summarized by Table 2. Please, consult [34] for full details regarding this benchmark set.

### Clustering methods

We employed four different clustering methods in our comparison, which are briefly reviewed in the sequel.

The k-medoids clustering method [25] is similar to the more popular k-means [63]. The only difference between these two clustering methods is due to the fact that, in k-medoids, each cluster is summarized by a medoid, i.e., a real object that minimizes its distance to all the remaining objects that belong to the cluster. The k-medoids method has three main steps: (i) for a given number *k *of clusters, *k *randomly chosen objects are selected as cluster medoids, (ii) each object in the dataset is assigned to the cluster with closest medoid and; (iii) cluster medoids are updated, i.e., for each cluster the new medoid is the object that has the lowest distance to the remaining objects that belong to its cluster. Steps (ii) and (iii) are repeated until a fixed number of iterations is exceeded or changes in clustering memberships are no longer observed. It is important to note that the k-medoids is not a deterministic method, i.e., for different initializations it may produce different outputs. To this extent, for each different dataset, number of clusters and distance adopted the method is initialized 50 times.

Hierarchical clustering methods are fairly common in the gene expression literature. We consider three different variants of agglomerative hierarchical clustering [23], i.e., Average-Linkage, Complete-Linkage and Single-Linkage. These methods take as input a proximity matrix generated from a dataset and produce as output a hierarchy of partitions, usually referred to as a dendrogram. Hierarchical clustering methods have two main steps: (i) each one of the objects is assigned to a singleton cluster, i.e., a cluster with a single object and; (ii) the two closest clusters are merged into a new cluster comprising their objects. Step (ii) is then repeated until a single cluster is obtained. Note that differences among Average-Linkage, Complete-Linkage and, Single-Linkage are defined by how the distance between clusters is computed, in order to identify the two closest clusters. For Average-Linkage this distance is given by the mean distance among all objects belonging to different clusters. For Complete-Linkage this distance is given by the farthest distance between objects in different clusters. In Single-Linkage it is provided by the smallest distance among objects belonging to different clusters. To obtain partitions with distinct cluster numbers we just have to "cut" the resulting dendrogram at the desired level.

Finally, the intervals $\left[2,\left\lceil \sqrt{o}\right\rceil \right]$, that comprehend the number of clusters considered during our second and third evaluation scenarios, are chosen due to its common usage in the clustering literature [64,65].

### Clustering validity

In the following we briefly describe the two *traditional *clustering validity criteria employed in order to assess the quality of partitions. Note that for gene time-series datasets we also employed a biologically driven validation methodology, as we already detailed.

#### Adjusted rand index

For cases in which a reference partition is available one can employ external validation measures to quantify the quality of the results. Due to its correction that takes into account conformities between partitions found by chance [66], we choose the Adjusted Rand [23,47], defined by Eq. (22), to evaluate clustering results. The greater its value, the greater is the concordance between the two partitions under comparison, with values close to 0 indicating conformities found by chance. Given a partition  U and a reference partition  V, in Eq. (22), (*a*) accounts for the total number of object pairs belonging to the same cluster in both  U and  V; (*b*) represents the total number of object pairs in the same cluster in  U and in different clusters in  V; (*c*) is the total number of object pairs that are in different clusters in  U and in the same cluster in  V; and (*d*) is the total number of object pairs that are in different clusters in both  U and  V.

(22) $$AR=\frac{a-\frac{\left(a+b\right)\left(a+c\right)}{\left(a+b+c+d\right)}}{\frac{\left(a+b\right)\left(a+c\right)}{2}-\frac{\left(a+b\right)\left(a+c\right)}{\left(a+b+c+d\right)}}$$

#### Silhouette index

To estimate the number of clusters in our third evaluation scenario, a relative index of comparison between partitions is also employed. The Silhouette index is defined by Eq. (23), considering a partitioning of *m *objects in *k *disjoint clusters. In Eq. (23), *u*(*i*) represents the average distance of **x **and all the remaining objects of its cluster. Value *v*(*i*) is obtained as follows: for a given object **x**, the average distance of **x **and all the objects from a given cluster is obtained. This process is repeated for all the *k − *1 clusters, excluding the cluster to which **x **belongs. At the end of the process the lowest mean value found is attributed to *v*(*i*). In other words, *v*(*i*) stands for the mean distance between **x **and its neighbor cluster (closest cluster). Silhouette, which is a maximization measure, has its values within [*−*1, 1].

(23) $$S=\frac{1}{m}\overset{m}{\underset{i=1}{\sum }}\frac{v\left(i\right)-u\left(i\right)}{max\left\{v\left(i\right),u\left(i\right)\right\}}$$

We choose the Silhouette based on its superior results in comparison to other relative criteria, as demonstrated by [49,67,68]. We also note that the Silhouette has already been successfully employed in order to estimate the number of cluster for gene expression data, e.g., [69-71].

Finally, we would like to note, that by using the Silhouette index we simulate a real application in which the user does not have any a priori information regarding the number of clusters present in the data. It is important to make clear, that the use of relative indexes (such as the Silhouette) is just part of the more general procedure that comprehends the whole clustering analysis, i.e., (i) pre-processing, (ii) clustering and, (iii) validation [72]. To this extent, in a real application, relative indexes may, in turn, help the user to choose the "best" partition or the "best" number of clusters for a given dataset (according to the criterion). For a review of clustering validation techniques in gene expression, please refer to [72].

### Friedman and Nemenyi statistical tests

Statistical tests were employed to assess the significance of the results obtained during our experimental evaluation. Based on the work of [73] we use Friedman [74] and Nemenyi [75] (with p-value = 0.05), given that they are more appropriate for evaluating the results of a collection of methods obtained over different datasets.

## Competing interests

The authors declare that they have no competing interests.

## Authors' contributions

PAJ implemented the methods and performed the experiments. PAJ, RJGBC and IGC designed the study, evaluated the results and wrote the manuscript. All authors read and approved the final manuscript.

## Declarations

The publication costs for this article were funded by Brazilian Research Agencies CAPES, CNPq, FACEPE and FAPESP (Processes #2011/04247-5 and #2012/15751-9). It was also partially funded by the Excellence Initiative of the German federal and state governments and the German Research Foundation through Grant GSC 111 and IZKF Aachen (Interdisciplinary Centre for Clinical Research within the faculty of Medicine at RWTH Aachen University).

This article has been published as part of *BMC Bioinformatics *Volume 15 Supplement 2, 2014: Selected articles from the Twelfth Asia Pacific Bioinformatics Conference (APBC 2014): Bioinformatics. The full contents of the supplement are available online at http://www.biomedcentral.com/bmcbioinformatics/supplements/15/S2.
